# A chest radiograph scoring system in patients with severe acute respiratory infection: a validation study

**DOI:** 10.1186/s12880-015-0103-y

**Published:** 2015-12-29

**Authors:** Emma Taylor, Kathryn Haven, Peter Reed, Ange Bissielo, Dave Harvey, Colin McArthur, Cameron Bringans, Simone Freundlich, R. Joan H. Ingram, David Perry, Francessa Wilson, David Milne, Lucy Modahl, Q. Sue Huang, Diane Gross, Marc-Alain Widdowson, Cameron C. Grant, On behalf of the SHIVERS investigation team

**Affiliations:** Starship Children’s Hospital, Auckland, New Zealand; The SHIVERS study, Auckland and Wellington, New Zealand; Children’s Research Centre, Starship Children’s Hospital, Auckland, New Zealand; Institute of Environmental Science and Research, Wellington, New Zealand; Department of Critical Care Medicine, Auckland City Hospital, Auckland, New Zealand; University of Auckland, Auckland, New Zealand; Infectious Diseases, Auckland City Hospital, Auckland, New Zealand; Radiology, Starship Children’s Hospital, Auckland, New Zealand; Radiology, Auckland City Hospital, Auckland, New Zealand; Centers for Disease Control and Prevention (CDC), Atlanta, USA; Department of Paediatrics: Child and Youth Health, Faculty of Medical and Health Sciences, The University of Auckland, Private Bag 92019, Wellesley Street, Auckland, 1142 New Zealand

**Keywords:** Influenza, Humans, Radiography, Thoracic, Respiratory tract infections, Validation studies

## Abstract

**Background:**

The term severe acute respiratory infection (SARI) encompasses a heterogeneous group of respiratory illnesses. Grading the severity of SARI is currently reliant on indirect disease severity measures such as respiratory and heart rate, and the need for oxygen or intensive care. With the lungs being the primary organ system involved in SARI, chest radiographs (CXRs) are potentially useful for describing disease severity. Our objective was to develop and validate a SARI CXR severity scoring system.

**Methods:**

We completed validation within an active SARI surveillance project, with SARI defined using the World Health Organization case definition of an acute respiratory infection with a history of fever, or measured fever of ≥ 38 °C; and cough; and with onset within the last 10 days; and requiring hospital admission. We randomly selected 250 SARI cases. Admission CXR findings were categorized as: 1 = normal; 2 = patchy atelectasis and/or hyperinflation and/or bronchial wall thickening; 3 = focal consolidation; 4 = multifocal consolidation; and 5 = diffuse alveolar changes.

Initially, four radiologists scored CXRs independently. Subsequently, a pediatrician, physician, two residents, two medical students, and a research nurse independently scored CXR reports. Inter-observer reliability was determined using a weighted Kappa (κ) for comparisons between radiologists; radiologists and clinicians; and clinicians. Agreement was defined as moderate (κ > 0.4–0.6), good (κ > 0.6–0.8) and very good (κ > 0.8–1.0).

**Results:**

Agreement between the two pediatric radiologists was very good (κ = 0.83, 95 % CI 0.65–1.00) and between the two adult radiologists was good (κ = 0.75, 95 % CI 0.57–0. 93).

Agreement of the clinicians with the radiologists was moderate-to-good (pediatrician:κ = 0.65; pediatric resident:κ = 0.69; physician:κ = 0.68; resident:κ = 0.67; research nurse:κ = 0.49, medical students: κ = 0.53 and κ = 0.56).

Agreement between clinicians was good-to-very good (pediatrician vs. physician:κ = 0.85; vs. pediatric resident:κ = 0.81; vs. medicine resident:κ = 0.76; vs. research nurse:κ = 0.75; vs. medical students:κ = 0.63 and 0.66).

Following review of discrepant CXR report scores by clinician pairs, κ values for radiologist-clinician agreement ranged from 0.59 to 0.70 and for clinician-clinician agreement from 0.97 to 0.99.

**Conclusions:**

This five-point CXR scoring tool, suitable for use in poorly- and well-resourced settings and by clinicians of varying experience levels, reliably describes SARI severity. The resulting numerical data enables epidemiological comparisons of SARI severity between different countries and settings.

## Background

Hospital-based surveillance for severe acute respiratory infection (SARI) has been implemented globally [1, 2]. The term SARI encompasses a heterogeneous group of respiratory illness syndromes. Clinical features of these syndromes overlap, with a broad spectrum of disease severity, ranging from overnight hospital admission to disease that causes death despite intensive care. Recent descriptions of SARI have highlighted this spectrum of severity and identified population subgroups at increased risk of severe or fatal disease, for example pregnant women, children with high-risk medical conditions, and individuals with diabetes or obesity [3–6].

However, grading the clinical severity of SARI in a manner that allows regional or temporal comparisons has proven difficult. Currently we are reliant on indirect disease severity measures, for example respiratory rate, presence of indrawing, hemoglobin oxygen saturation, use of oxygen, intensive care unit admission and length of hospitalization. The availability of such measures varies extensively worldwide due to differences in healthcare-seeking behavior, criteria for hospitalization, the length of hospitalization, and availability of oxygen and intensive care. In both developing and developed countries, chest radiographs are one of the more standardized pieces of data collected in epidemiological studies of acute respiratory infections [7]. For example, chest radiographs are a component of the contemporary global epidemiological study of severe pneumonia in children: the Pneumonia Etiology Research for Child Health project, being conducted in South Africa, Zambia, Kenya, the Gambia, Mali, Thailand, and Bangladesh [8].

Included among the 15 recommendations made by the World Health Organization (WHO) from their review of the functioning of the 2005 International Health Regulations in relation to the 2009 H1N1 pandemic, was the need to develop and apply measures to assess the severity of a pandemic beyond the number of cases and deaths [9]. With the lungs being the primary organ system involved in SARI, chest radiographs (CXRs) are potentially useful for describing disease severity. Clinically, physicians use CXRs to define the extent of lung involvement and the presence of pulmonary complications, yet currently our ability to utilize this information to define SARI severity or to compare severity between population subgroups, is limited.

To date, the use of CXRs has focused primarily on the diagnosis of specific syndromes, in particular pneumonia, and in informing therapeutic decisions for individual patients, for example antibiotic treatment. This is despite poor inter-observer agreement in use of a CXR to determine the presence of pneumonia or to indicate a bacterial versus a viral etiology (Kappa values of 0.46 for pneumonia and 0.27–0.38 for viral vs. bacterial etiology) [10–12].

In an attempt to improve the consistency of CXR interpretation between epidemiological studies, the WHO standardized the interpretation of CXRs for the diagnosis of pneumonia in children [7]. However, the utility of WHO’s CXR assessment method, when applied to clinical studies of SARI, has been questioned due to a low sensitivity for diagnosing pneumonia [13]. Additionally, there are inherent limitations in assessing severity with a tool that dichotomizes CXRs only on whether or not alveolar consolidation is present.

Case series of SARI from the recent H1N1 influenza pandemic have provided a more complete description of the specific associated radiological features, which include rapidity of progression, broad regions of affected lung, extensive infiltrates, and ground glass changes [14–17]. Being able to quantify the extent of radiographic changes in a systematic manner would allow for better and more objective assessment and comparison of rates and severity of SARI between studies, over time, and in different global regions; and potentially enable a better understanding of prognosis.

For large surveillance projects, a CXR scoring system usually cannot demand significant additional resources, such as independent specialist radiologists to review all CXRs. One potential approach is to utilize the information contained in the report of the CXR that a radiologist produces as a component of a patient’s hospital medical record, allowing non-radiologist clinicians to use this information to assess severity. However, because of the individual variability in how different radiologists report CXRs, an assessment of the validity of this approach is necessary.

Our objective was to assess the validity of a standardized scoring system for reviewing CXR reports in patients hospitalized with SARI. If valid, such an approach would have the potential to allow the inclusion of CXR data in epidemiological studies of SARI worldwide, in a cost- and time-efficient manner, and form an integral part of overall severity assessment for seasonal and pandemic influenza.

## Methods

### Study design and setting

**Fig. 1:**
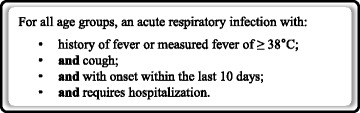
World Health Organization severe acute respiratory infection case definition [2]

We described the CXR abnormalities of a case series of children and adults hospitalized with a SARI, as defined by the WHO (Fig. 1) [2]. We identified SARI cases by active surveillance within a geographically defined region of Auckland, New Zealand (latitude 36°S). This active surveillance is a component of the Southern Hemisphere Influenza Vaccine Effectiveness Research and Surveillance (SHIVERS) project [18]. We obtained ethical approval from the Northern A Health and Disability Ethics Committee (NTX/11/11/102 AM02). The Ethics committee considered written consent unnecessary for this collection of non-sensitive data from routine in-hospital clinical management and diagnostic testing. We obtained verbal consent from all participants or, in the case of minors, from their caregivers. Verbal explanation of the reason for collection of this additional information and its use was given to each patient, consistent with the New Zealand Code of Health and Disability Services Consumers’ Rights (Right 6: Right to be fully informed) [19].

SHIVERS surveillance includes that of all hospitalizations with SARI to the four hospitals in the study region (Auckland City Hospital, Middlemore Hospital, Starship Children’s Hospital, and Kidz First Children’s Hospital) since May 1, 2012. The population in this region (*n* = 905,634), as defined at the 2013 national census, is diverse with respect to ethnicity (25 % Asian, 16 % Pacific, 11 % Māori, 47 % European and other) and socioeconomic status (20 % of sample in the least deprived quintile of households, 27 % in the most deprived quintile) [18].

For this CXR severity scoring validation study, we identified a case series of 250 people with SARI. From the 926 SARI cases identified during the surveillance period at two of the SHIVERS surveillance hospitals, Auckland City and Starship Children’s Hospital, we stratified the data into two age groups, 0 to 14 years (children), and 15 years and over (adults). Using random numbers, we randomly selected 125 children and 125 adults with SARI. To be eligible both a CXR and a nasopharyngeal sample had to have been collected. If a SARI case had more than one CXR recorded, only the first CXR was included.

### Chest radiograph scoring

**Fig. 2:**
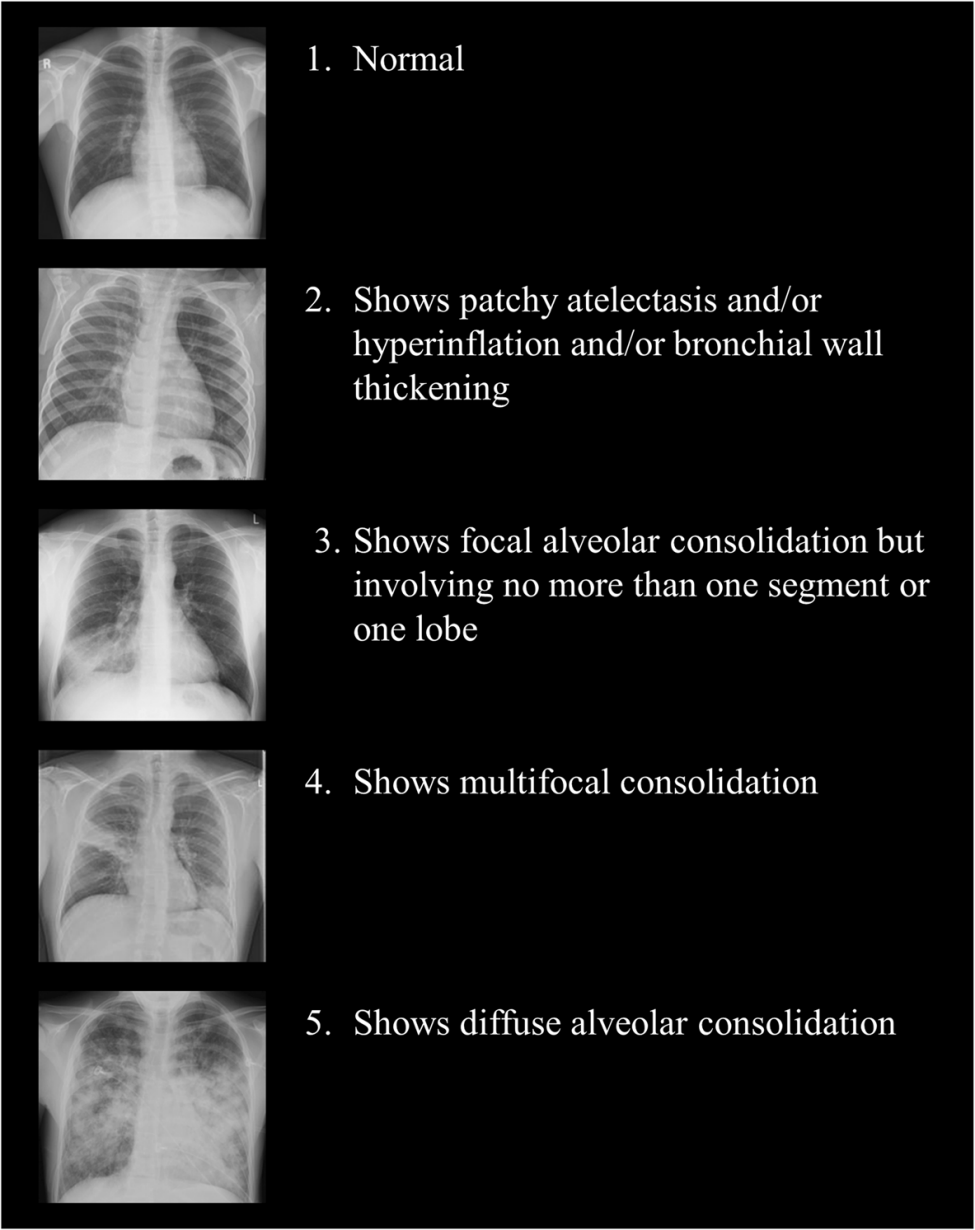
The chest radiograph severity scoring system

Following a literature review, and with input from pediatricians, adult physicians and intensivists working within the SHIVERS project, we devised a five-point CXR scoring tool to record the severity of lung abnormalities (Fig. 2).

### Scoring of chest radiograph images by study radiologists

For the purposes of this study, two radiologists with expertise in interpreting pediatric chest radiographs reviewed the 125 chest radiographs of the pediatric patients and two radiologists with expertise in interpreting adult chest radiographs reviewed the 125 chest radiographs of adult patients. These four radiologists formed the ‘study radiologist’ team. The two pediatric and two adult study radiologists, blinded to the clinical details of each case, independently read and assigned a score of 1 to 5 for the 125 CXR images from children aged 0–14 years and the 125 CXR images from adults aged 15 years and over.

### Reading of chest radiographs by clinical radiologists

Clinical radiologists based at the two hospitals, who were unaware of this validation study, read the CXR of each participant as a component of routine clinical care and then entered a written report of their reading of the CXR into the patient’s medical record.

### Scoring of the clinical radiologists’ chest radiograph reports by clinicians

For the purposes of this study, we created a clinician team that included a pediatrician, an internal medicine physician, a pediatric resident, an internal medicine resident, two medical students and a research nurse. Each member of this clinician team independently read all 250 CXR reports written by the clinical radiologists and, using the same scoring system as had been applied by the four study radiologists, assigned a score of 1 to 5 for each report (actual radiographs were not reviewed). Clinicians with considerable experience (attending physicians and residents), as well as those with more limited experience (medical students and a research nurse), in reading CXR reports performed the CXR severity scoring.

We compared the clinicians’ scoring of the clinical radiologists’ chest radiograph report with the reference scores from the study radiologists’ reading of the chest radiographs. We also compared clinicians’ scores with one another, using the pediatrician’s scores as the clinician reference standard.

### Agreement within and between radiologists and clinicians on chest radiograph scoring

A weighted Kappa (κ) score (weighted by 1-[(i-j)/(1-k)]^2^) was used to assessment agreement on chest radiograph scoring. We compared the scores of, and determined the agreement between, each pair of study radiologists. Then, using the most senior of each radiologist pair (pediatric and adult) as the reference standard, we compared the CXR report scoring of each clinician with the study radiologist’s score.

Agreement between pairs of study radiologists, of each clinician with the study radiologists score, and between pairs of clinicians were then determined. Weighted Kappa scores and 95 % confidence intervals (CI) were calculated using StatsDirect statistical software version 2.7.9 (Altrincham, Cheshire, UK). We used a weighted rather than raw Kappa score as this adjusts for the degree of disagreement when the compared categories are ordinal. Weighted Kappa scores were defined as showing ‘poor’ (κ ≤ 0.2), ‘fair’ (>0.2 to 0.4), ‘moderate’ (>0.4 to 0.6), ‘good’ (>0.6 to 0.8) or ‘very good’ (>0.8 to 1.0) agreement [20].

Following completion of the scoring and identification of the CXR reports with discrepant scores, the pediatrician then met individually with each of the other clinicians to determine if we could achieve a consensus severity score for these reports. We then recalculated the κ scores for the radiologist-clinician and clinician-clinician comparisons.

### Sample-size estimates

We based sample-size estimates upon the five-point ordinal scale scoring system, assuming a distribution of scores of 10 %, 25 %, 25 %, 25 %, and 15 % across the five categories. For a sample-size of 200, the Cohen’s Kappa measure of agreement will have a confidence interval in the order of ±0.14 (assuming 100 % agreement and weighting to allow for the ordinal nature of scores). Given the potential for the actual distributions across categories to differ from these assumptions we increased the sample-size to 250.

## Results

### Study sample demographics, clinical illness, respiratory viral isolates and CXR abnormalities (Table 1)

**Table 1:** Demographic, clinical, and respiratory viral characteristics, and discharge diagnoses of random sample of 250 patients hospitalized with a severe acute respiratory infection and identified by active surveillance

	Children	Adults
Variable	(n_1_ = 125)	(n_2_ = 125)
Demographics		
Age in years, median (IQR^a^)	1 (0–3)	60 (42–75)
Male gender, *n* (%)	70 (56)	66 (53)
Ethnicity, *n* (%)		
European and other	56 (45)	75 (60)
Maori	22 (17)	13 (10)
Pacific	36 (29)	19 (15)
Asian	11 (9)	18 (15)
Self-defined health^b^, *n* (%)		
Excellent	51 (42)	11 (9)
Very good	32 (26)	39 (32)
Good	25 (20)	44 (36)
Fair	5 (4)	20 (16)
Poor	10 (8)	9 (7)
Smoking history (adults only)		
Ever smoker, *n* (%)	-	65 (50)
Current smoker, *n* (%)	-	18 (14)
Clinical features of SARI illness		
Presenting syndrome^c^, *n* (%)		
Suspected acute upper respiratory tract infection	6 (5)	3 (3)
Suspected croup	4 (3)	0 (0)
Suspected bronchiolitis	42 (36)	0 (0)
Suspected pneumonia	50 (42)	47 (39)
Exacerbation of adult chronic lung disease	0 (0)	11 (9)
Exacerbation of asthma	7 (6)	7 (6)
Exacerbation of childhood chronic lung disease	1 (1)	0 (0)
Respiratory failure	0 (0)	3 (3)
Febrile illness with respiratory symptoms	3 (3)	30 (25)
Other suspected acute respiratory infection	5 (4)	18 (15)
Length of stay in days, median (IQR^a^)	3 (2–5)	3 (2–6)
Intensive care unit admission, *n* (%)	21 (17)	4 (3)
Respiratory viral testing and results		
Influenza virus identified^d^, *n* (%)	26 (21)	31 (25)
Non-influenza respiratory virus identified^e^, *n* (%)	80 (81)	27 (25)
Discharge diagnosis category^f^		
Respiratory	119 (95)	93 (74)
Cardiovascular	0 (0)	6 (5)
Infectious diseases	4 (3)	6 (5)
Other organ systems	2 (2)	20 (16)

^*^IQR = interquartile range

^b^n_1_ = 123, n_2_ = 123

^c^n_1_ = 118, n_2_ = 119. Suspected upper respiratory tract infection includes coryza and pharyngitis; exacerbation of adult chronic lung disease includes chronic obstructive lung disease, emphysema, and bronchitis; exacerbation of childhood chronic lung disease includes bronchiectasis and cystic fibrosis; febrile illness with respiratory symptoms includes shortness of breath

^d^n_1_ = 125, n_2_ = 124. Child: influenza A (H1N1)pdm09 *n* = 7, influenza A (H3N2) *n* = 9, influenza A (not subtyped) *n* = 1, influenza B *n* = 9; Adult: influenza A (H1N1)pdm09 *n* = 8, influenza A (H3N2) *n* = 12, influenza A (not subtyped) *n* = 5, influenza B *n* = 6

^e^n_1_ = 99, n_2_ = 109. Child: respiratory syncytial virus *n* = 49, rhinovirus *n* = 24, parainfluenza virus *n* = 3, adenovirus *n* = 13, human metapneumovirus *n* = 5; Adult: respiratory syncytial virus *n* = 7, rhinovirus *n* = 13, parainfluenza virus *n* = 2, adenovirus *n* = 0, human metapneumovirus *n* = 4

^f^Based upon ICD principal discharge diagnosis codes

**Fig. 3:**
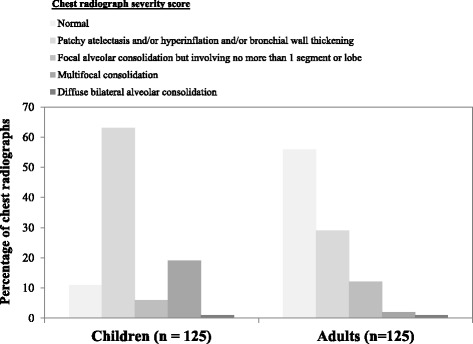
Distribution of radiologist’s chest radiograph scores for children and adults hospitalized with a serious acute respiratory infection

The median (interquartile range) age of the children with SARI was 1 (0–3) year of age and of the adults was 60 (42–75) years of age. Sixty-five (50 %) of the adults were smokers, of whom 18 (28 %) were current smokers. The most common presenting syndromes among the children were suspected pneumonia (42 %) and suspected bronchiolitis (36 %), and among the adults were suspected pneumonia (39 %) and febrile illness with respiratory symptoms (25 %). Median length of hospital stay for children and adults was 3 days. Ten percent (children 17 %, adults 3 %) required intensive care. Laboratory testing identified influenza viruses in 23 % of SARI cases and non-influenza respiratory viruses (respiratory syncytial virus, rhinovirus, parainfluenza virus types 1–3, adenovirus, or human metapneumovirus) in 43 %. In 12 (10 %) children and one (1 %) adult co-detection of influenza and a non-influenza virus occurred. The proportion of SARI cases that were influenza positive was similar for children versus adults (21 % vs. 25 %, *P* = 0.43). A larger proportion of the SARI cases in children, compared to adults, were positive for non-influenza respiratory viruses (81 % vs. 25 %, *P* < 0.001). A larger proportion of the SARI cases in children, compared to adults were assigned a principal discharge diagnosis code for a respiratory illness (95 % vs. 74 %, *P* < 0.001). The distribution of CXR scores across the five scoring categories differed between children and adults (*P* < 0.001; Fig. 3).

### Chest radiograph scoring agreement

#### Radiologist with radiologist agreement

Agreement within pairs of radiologists who scored the radiographs was ‘very good’ for the pediatric radiologists (κ = 0.83) and ‘good’ for the adult radiologists (κ = 0.75) (Table 2).

**Table 2:** Agreement between radiologists in scoring severe acute respiratory infection CXRs from their reading of the digital CXR images and agreement in scoring severe acute respiratory infection CXRs: clinicians reading of CXR reports versus radiologists reading of CXRs

	Weighted Kappa	Strength of
Health professional	(95 % CI)	agreement^a^
Radiologist Agreement		
Pediatric radiologists	0.83 (0.65 to 1.00)	‘Very good’
Adult radiologists	0.75 (0.57 to 0.93)	‘Good’
Radiologist-clinician agreement		
Radiologist vs. pediatrician	0.65 (0.52 to 0.78)	‘Good’
Radiologist vs. internal medicine physician	0.68 (0.55 to 0.80)	‘Good’
Radiologist vs. internal medicine resident	0.66 (0.53 to 0.78)	‘Good’
Radiologist vs. pediatric resident	0.69 (0.56 to 0.82)	‘Good’
Radiologist vs. medical student 1	0.56 (0.44 to 0.69)	‘Moderate’
Radiologist vs. medical student 2	0.53 (0.40 to 0.66)	‘Moderate’
Radiologist vs. research nurse	0.49 (0.36 to 0.62)	‘Moderate’

^a^Agreement: weighted Kappa <0.2 = ‘poor’, >0.2 to 0.4 = ‘fair’, >0.4 to 0.6 = ‘moderate’, >0.6 to 0.8 = ‘good’, >0.8 to 1.0 = ‘very good’ agreement

CI = confidence interval

#### Clinician with radiologist agreement

The κ values for agreement of clinicians with radiologists ranged from 0.49 to 0.69. Agreement of the clinician’s scoring of the CXR reports with the senior radiologists scoring the CXR images was ‘good’ for the pediatrician (κ = 0.65), internal medicine physician (κ = 0.68), internal medicine resident (κ = 0.66), and pediatric resident (κ = 0.69); and ‘moderate’ for the two medical students (κ = 0.53 and 0.56) and the research nurse (κ = 0.49) (Table 2).

#### Clinician with clinician agreement

The κ values for agreement between clinician pairs ranged from 0.63 to 0.85. Agreement between clinician pairs of their CXR report scoring was ‘very good’ for the pediatrician versus the internal medicine physician (κ = 0.85) and the pediatrician versus the pediatric resident (κ = 0.81); and ‘good’ for comparisons between the pediatrician and the internal medicine resident (κ = 0.77), medical students (κ = 0.63 and 0.66), and research nurse (κ = 0.75) (Table 3).

**Table 3:** Agreement between clinician pairs in classification of CXR abnormalities in patients with a severe acute respiratory infection

			Number of CXRs with discrepant scores
	Weighted Kappa	Strength of	*n* = 250
Clinician-clinician combination	(95 % CI)	agreement^a^	*n* (%)
Agreement after independent review			
Pediatrician vs. internal medicine physician	0.85 (0.73 to 0.98)	‘Very good’	39 (16)
Pediatrician vs. internal medicine resident	0.76 (0.63 to 0.88)	‘Good’	48 (19)
Pediatrician vs. pediatric resident	0.81 (0.68 to 0.95)	‘Very good’	51 (20)
Pediatrician vs. medical student 1	0.66 (0.53 to 0.78)	‘Good’	67 (27)
Pediatrician vs. medical student 2	0.63 (0.50 to 0.76)	‘Good’	70 (28)
Pediatrician vs. research nurse	0.75 (0.62 to 0.88)	‘Good’	56 (22)
Agreement after combined review of CXRs with discrepant scores			
Pediatrician vs. internal medicine physician	0.98 (0.90 to 1.06)	‘Very good’	3 (1)
Pediatrician vs. internal medicine resident	0.99 (0.87 to 1.12)	‘Very good’	4 (2)
Pediatrician vs. pediatric resident	0.97 (0.84 to 1.09)	‘Very good’	5 (2)
Pediatrician vs. medical student 1	0.99 (0.86 to 1.11)	‘Very good’	3 (1)
Pediatrician vs. medical student 2	0.98 (0.85 to 1.10)	‘Very good’	3 (1)
Pediatrician vs. research nurse	0.99 (0.86 to 1.11)	‘Very good’	6 (2)

^a^Agreement: weighted Kappa ≤0.2 = ‘poor’, >0.2 to 0.4 = ‘fair’, >0.4 to 0.6 = ‘moderate’, >0.6 to 0.8 = ‘good’, >0.8 to 1.0 = ‘very good’ agreement

*CI* Confidence interval

#### Clinician-radiologist agreement and clinician-clinician agreement following clinician review of chest radiographs with scoring discrepancies

Following review by clinician pairs of the CXR reports for which their scores were discrepant, and determination of whether a consensus score was possible, we recalculated radiologist-clinician and clinician-clinician agreement. The radiologist-clinician κ values ranged from 0.59 to 0.70 following this second CXR report review. The changes in κ values for agreement of the clinicians’ scoring with the radiologists’ scoring following this second CXR report review were smaller for the pediatrician (+3), internal medicine physician (−1), internal medicine resident (−3) and pediatric resident (+1) and larger for the medical students (+9, +14) and research nurse (+10) (Table 4). Agreement on CXR report scoring for all pairs of clinicians following this consensus meeting was ‘very good’ with κ scores ranging from 0.97 to 0.99 (Table 3).

**Table 4:** Agreement in classification of CXR abnormalities in patients with a severe acute respiratory infection: clinicians reading of CXR reports following clinician-clinician review of discrepant scores versus radiologists reading of CXRs

	Weighted Kappa	Strength of
Radiologist-clinician combination	(95 % CI)	agreement^a^
Radiologist vs. pediatrician	0.68 (0.60 to 0.76)	‘Good’
Radiologist vs. internal medicine physician	0.67 (0.59 to 0.76)	‘Good’
Radiologist vs. adult medical resident	0.65 (0.56 to 0.74)	‘Good’
Radiologist vs. pediatric medical resident	0.70 (0.62 to 0.78)	‘Good’
Radiologist vs. medical student 1	0.65 (0.56 to 0.74)	‘Good’
Radiologist vs. medical student 2	0.67 (0.59 to 0.76)	‘Good’
Radiologist vs. research nurse	0.59 (0.48 to 0.69)	‘Moderate’

^a^Agreement: weighted Kappa ≤0.2 = ‘poor’, >0.2 to 0.4 = ‘fair’, >0.4 to 0.6 = ‘moderate’, >0.6 to 0.8 = ‘good’, >0.8 to 1.0 = ‘very good’ agreement

*CI* Confidence interval

The distribution of CXR scores skewed more to the lower (more normal) scores than was anticipated in the study sample-size calculation. However, across all comparisons the κ estimates had average confidence intervals of ± 0.12 (range ± 0.08 to ± 0.18), which was in keeping with our sample-size estimate.

## Discussion

Using a novel five-point ordinal scoring system, we described the agreement of clinicians with radiologists and between clinicians in the interpretation of CXR abnormalities in patients with SARI. We observed ‘good’ to ‘very good’ inter-observer agreement between radiologists who reviewed the original radiographs and applied the scoring system. Agreement between radiologists and of radiologists with clinicians was ‘moderate’ to ‘very good’. Inter-observer agreement between clinicians of various levels of experience was ‘good’ to ‘very good’. Following a consensus review by clinician pairs of radiograph reports with discrepant scores, clinician agreement with the radiologists improved for the clinicians who were less experienced in CXR interpretation and agreement between all clinician pairs became ‘very good’.

Our study used data collected from 250 prospectively enrolled SARI cases (125 pediatric; 125 adult) selected randomly from a larger number of SARI cases identified by active surveillance within a defined region and study period. The CXR’s from the children were more severely abnormal compared to those obtained from adults. The largest differences in comparisons between the CXR’s from children and adults were in the proportion with CXR severity scores of 1 ‘normal’ (pediatric 11 %, adult 56 %) and of 2 ‘shows patchy atelectasis and/or hyperinflation and/or bronchial wall thickening’ (pediatric 63 %, adult 29 %). We postulate that this is due to age-related differences in lung anatomy, with young children having smaller airways with increased airway resistance; less alveoli and reduced alveolar surface area; and a more elastic and compliant chest wall compared to adults [21].

For large epidemiological studies of SARI, interpretation of CXRs by radiologists is both costly and time consuming. In contrast with the storage and subsequent review of a digital CXR image, a CXR report can be stored as a simple text document and read without the requirement for sophisticated software. Our approach allows inclusion of CXR data into a numerical data set without the need for complex methods, such as digital algorithms. Clinical personnel with less specialist training may use this approach and achieve acceptable levels of agreement with radiologists and with more experienced clinicians, especially if the opportunity for reviewing reports with discrepant scores is included.

A potential weakness of our study is that we have compared radiologists’ scores from the original CXR images to clinicians’ scores of the corresponding reports. However, we felt it was important to show the scoring tool was valid when applied to CXRs by radiologists, given that their interpretation is the gold standard. Establishing that there was agreement between radiologists was a necessary first step before proceeding with comparisons between radiologists and clinicians.

With few admissions to intensive care and very few CXRs with scores of ‘5’, we cannot be sure agreement for extremely abnormal CXRs is high. However, most of the disagreement in previously reported studies is in the mid-range of our categorization system, e.g. the differentiation between bronchiolitis and pneumonia in children [11]. Our validation was limited to the first CXR of the hospital admission so may not have included the most abnormal radiograph from each patient. However, for surveillance this is most appropriate.

Application of this CXR scoring system allows for an evaluation of the relationship between the severity of CXR abnormalities and exposure to factors that potentially prevent respiratory disease, for example, the pneumococcal conjugate vaccine [22]. It also allows for an evaluation of the relationship between the severity of CXR changes on hospital admission and subsequent health care utilization, for example, intensive care unit admission. Being able to include numerical data that describes the severity of chest radiograph abnormalities may allow for increased precision in the application of tools that use vital sign and laboratory abnormalities to assist in clinical decision making in patients with SARI [23, 24].

Our scoring system is relatively simple and simpler, for example, to the approach developed for the scoring of CXRs from patients with chronic respiratory conditions such as cystic fibrosis [25]. Given that we were describing an acute respiratory illness we specifically excluded a description of the presence of chronic disease, non-respiratory disease and/or complications from this validation study. We believe that separate description and recording of such abnormalities and of the acute changes related to SARI is more appropriate.

The inter-observer agreements achieved in this study were ‘moderate’ (κ <0.4 to 0.6) to ‘very good’ (κ >0.8 to 1.0). This is an acceptable result when compared to other studies that have examined inter-observer reliability in assessing CXRs, for example in adult community-acquired pneumonia, where κ values were less than 0.50 [10–12]. The inter-observer agreements achieved in this study also compare favorably with those found when using the WHO criteria for radiologically confirmed pneumonia in children to examine the efficacy of pneumococcal conjugate vaccines in preventing pneumonia (Kappa = 0.58) [22]. We postulate that better agreement was reached with our scoring system because it only required a description of the presence of abnormalities rather than an interpretation of whether or not these changes identified a specific syndrome, for example pneumonia or bronchiolitis [26]. Poor agreement between clinicians on the finer details of chest radiograph interpretation is evident in studies of both adults and children with community-acquired pneumonia [26, 27].

Consistent with the published literature, agreement of clinicians with radiologists and agreement between clinicians varied with the level of clinician experience in reading CXR reports [7, 27–29]. Including clinicians with less clinical experience in CXR interpretation (medical students and research nurse) and demonstrating that they could reach reasonable levels of agreement compared with more experienced colleagues, shows promise for the application of this tool by such members of research teams.

Our sample was too small to allow us to reliably investigate or describe the chest radiographic features of population subgroups defined, for example by presenting syndrome, intensity of care required, or respiratory viruses detected. The first two years of SHIVERS surveillance identified more than 3,500 cases of SARI. As we plan for five years of surveillance within the SHIVERS project, we anticipate that we will have sufficient study power to complete these important subgroup analyses and will now be able to include this measure of CXR severity in our analyses.

## Conclusions

Our CXR report scoring tool provides a reliable and valid method for describing the overall severity of acute radiographic abnormalities in patients with SARI. This low-tech, simple, and relatively quick method makes use of existing information. The information recorded can easily be included in numerical data sets without requiring transformation via more complex methods. We have shown that the interpretation of a pre-existing written radiologist report is an appropriate proxy for film interpretation. To our knowledge, no previous evaluation of this approach exists. Our study demonstrates that clinicians with diverse levels of training and experience can reach adequate agreement when scoring the severity of CXR abnormalities in SARI. We anticipate that this scoring tool will facilitate a clear description of the respiratory characteristics of SARI and will enable epidemiological comparisons between SARI populations from different countries and settings.

## Availability of supporting data

Study data is available from the corresponding author.

## Abbreviations

CXR: Chest radiograph
SARI: Severe acute respiratory infection
SHIVERS: Southern hemisphere influenza vaccine effectiveness research and surveillance
